# Overexpression of OLC1 Promotes Tumorigenesis of Human Esophageal Squamous Cell Carcinoma

**DOI:** 10.1371/journal.pone.0090958

**Published:** 2014-03-07

**Authors:** Xiao Li, Jing Suo, Shujuan Shao, Liyan Xue, Wei Chen, Lijia Dong, Ji Shi, Ming Fu, Ning Lu, Qimin Zhan, Tong Tong

**Affiliations:** 1 State Key Laboratory of Molecular Oncology, Cancer Institute & Cancer Hospital, Chinese Academy of Medical Sciences & Peking Union Medical College, Beijing, China; 2 Department of Pathology, Cancer Institute & Cancer Hospital, Chinese Academy of Medical Sciences & Peking Union Medical College, Beijing, China; 3 CAS Key Laboratory of Separation Science for Analytical Chemistry, Dalian Institute of Chemical Physics, Chinese Academy of Science, Dalian, Liaoning, China; 4 Department of Histology and Embryology, Dalian Medical University, Dalian, Liaoning, China; The University of Hong Kong, China

## Abstract

**Purpose:**

OLC1 was recently identified to be a potential oncogene. However, the role of OLC1 in human esophageal cell carcinoma (ESCC) is unknown. The aim of this study was therefore to evaluate the expression of OLC1 in human ESCC from normal, premalignant, and malignant lesions, and to clarify the mechanisms by which OLC1 contributes to the progression of ESCC**.**

**Experimental Design:**

Two hundred and fourteen paired ESCC specimens, and an independent set from 28 ESCC patients, were used to analyze the correlation between OLC1 expression and the pathological characteristics of tumors using immunohistochemistry. Stable OLC1-overexpressing and OLC1-interfering esophageal cancer cells were established and a series of experimental methods were used to investigate the biological functions and mechanisms of action of OLC1.

**Results:**

We showed that OLC1 was overexpressed in 145 of 214 (67.8%) of human ESCC specimens, compared with in only 59 of 214 (27.57%) paired adjacent normal tissues (*P*<0.001). OLC1 overexpression occurred at a rate of 35% (10/28) at the stage of mild/moderate dysplasia, but was significantly upregulated to 66% (22/33) at the stages of severe dysplasia and *in situ* carcinoma, while 71% positive staining (22/28) was observed in invasive carcinoma tissues compared with normal tissues (*P*<0.05). We also provided evidence that OLC1 abnormalities significantly altered the cell proliferation and apoptosis induced by cytotoxic agents. OLC1 overexpression suppressed apoptosis, and was associated with attenuated caspase-3 activation and increased Bcl-2 stability.

**Conclusion:**

Our study provides strong evidence suggesting OLC1 abnormalities may contribute to the development of human ESCC and have some important clinical significance.

## Introduction

Human esophageal carcinoma is one of the most common malignant tumors worldwide, and 90% of cases are esophageal squamous cell carcinomas (ESCC). Compared with Western countries, cases of ESCC in the People’s Republic of China are characterized by a higher incidence and mortality rate. Several studies investigating ESCC pathophysiology have focused on changes in protein expression, which can contribute to the discovery of new biomarkers and help to develop novel strategies for the early diagnosis and individualized treatment of ESCC [1]. It was previously reported that p53 [2]–[4], Cyclin D1 [5]–[7], E-cadherin [8], K-ras [9], and VEGF [10]–[13] have altered expression in ESCC, suggesting that abnormalities in the expression of oncogenes and tumor suppressor genes contributes to esophageal carcinogenesis and malignant development. These abnormally expressed proteins may therefore serve as potential biomarkers for ESCC. We have recently demonstrated that Aurora-A and cyclin B1, which are key regulators of cell cycle progression, are overexpressed in patients with ESCC, and are associated with increased tumor invasion or metastasis. These findings suggest that Aurora-A and cyclin B1 may therefore serve as useful biomarkers for evaluating advanced ESCC and determining disease prognosis [14]–[18].

OLC1 was recently identified to be a candidate oncogene. It was originally identified by Yuan et al. [19] as one of fifty uncharacterized genes that were differentially expressed in squamous cell lung tumors compared with normal bronchial epithelial cells, and they named it OLC1. Recent studies from Yuan et al. suggest that OLC1 is overexpressed in human lung cancer, which may be linked to activation of the NF-κB pathway. In addition, a large-scale screening study of human genes has revealed that OLC1 can activate the MAPK signaling pathway [20].

The OLC1 gene is also known as KIAA0174, and its accession number is AK057902. OLC1 gene spans 33.47 kb on human chromosome 16q22.2, encoding the OLC1 protein that consists of 364 amino acids. This gene was first identified in non-lethal yeast mutants, and was named the Increased Sodium Tolerance 1 (IST1) gene [21]. The gene product of OLC1/IST1 is highly conserved from humans to yeast. In yeast, IST1 regulates the disassembly of endosomal sorting complexes, based on subcellular localization and the identification of specific protein-binding partners [22]–[25]. To our knowledge, few studies reporting cancer-related activities of OLC1 were made until recently. Because it is highly conserved, studying the roles of OLC1 could provide more insights into elucidating its biological role in human tumorigenesis.

The role of OLC1 in human esophageal carcinoma remains unknown. In this study, we evaluated the expression of OLC1 in human ESCC at different stages of tumorigenesis, from normal, premalignant, and malignant lesions. We also established stable OLC1-overexpressing and OLC1-interfering cell lines, based on human esophageal cancer cell lines, to investigate the biological functions of OLC1. We identified relationships between the dysregulation of OLC1 and the effects of cytotoxic agents on cell proliferation and apoptosis. Our study provides strong evidence suggesting that OLC1 may contribute to the development of esophageal cancer.

## Materials and Methods

### Cell Lines and Cell Culture

Human ESCC Cell lines were grown in RPMI 1640 medium (Invitrogen) supplemented with 10% fetal bovine serum, and maintained at 37°C in a humidified 5% CO_2_ incubator. The human EC9706, KYSE510, KYSE180, KYSE450 and KYSE150 were the origin of gifted cell lines indicated in our previous published references [15]–[18].

### Plasmids, Cell Transfections, and the Establishment of Stable Cell Lines

The plasmids pEGFP-N1, pEGFP-N1-OLC1, PGCsi-U6/neo/GFP-control and PGCsi-U6/neo/GFP-OLC1 were all kind gifts from Prof. Shujun Cheng (Chinese Academy of Medical Sciences & Peking Union Medical College, Beijing, China). Plasmid transfection was carried out using Lipofectamine 2000 (Invitrogen) according to the manufacturer’s instructions, and transfected cells were selected with G418 at 400 µg/mL (Geneticin sulfate, Gibco). For each transfection, colonies were trypsinized and collected to produce stable cell pools. The OLC1 stable cell lines were validated by western blot analysis, RT-PCR, and GFP fluorescence using fluorescence microscopy (Olympus).

### Patients and Tissue Samples

All specimens used for the study were obtained from the paraffin-embedded tissue archives of our Department of Pathology, as indicated in our previous published references [14], [15]. This study was approved by the ethics committee of Cancer Institute and Hospital, Chinese Academy of Medical Sciences (approval number: 12-71/605. issued date: July 26, 2012). All cancer patients in our Hospital were signed the clinical informed consent of surgical treatment. The tumor specimens resected were allowed for further pathological diagnosis and research program approved by the ethics committee of Cancer Institute and Hospital, Chinese Academy of Medical Sciences.

All 214 formalin-fixed, paraffin-embedded specimens were obtained from patients who were diagnosed with ESCC between 1999 and 2002. None of the patients had received radiotherapy or chemotherapy before surgery. All the cases were T3 stage with invasion to adventitia, and none had shown evidence of distance metastases (M0). Among these patients, 110 were T3N1M0 with lymph-node metastases, and 104 were T3N0M0 without lymph-node metastases, with a gender distribution of 172 male to 42 female. The age of patients ranged from 31 to 81 years, with a median of 58.87. The 214-paired ESCC tissues were used to prepare tissue microarrays, which were then used to analyze the correlation of OLC1 expression with the malignant features of the tumors using immunohistochemistry (IHC).

An independent set of paraffin-embedded tissue sections from 28 ESCC patients were used to verify the correlation between OLC1 protein expression and the stages of tumorigenesis of human ESCC. Twenty-three of these samples were from males, while five were from females, and the age of the patients ranged from 33 to 69 years-of-age. These samples included 25 normal adjacent, 28 mild to moderate dysplasia, 33 severe dysplasia/carcinoma in situ (CIS), and 28 invasive carcinoma tissues.

Two pathologists independently diagnosed all samples. One section was stained with hematoxylin and eosin (H&E) for histological evaluation, and the others were used for IHC analysis.

### Immunohistochemical Staining for Evaluating OLC1 Protein Expression in ESCC

The tissue sections were deparaffinized in xylene, and rehydrated in graded ethanol. After antigen retrieval with sodium citrate, sections were blocked with 1.5% normal blocking serum in PBS for 1-hr at room temperature. They were then incubated with a 1∶100 dilution of the primary anti-OLC1 antibody (provided by Prof. Shujun Cheng) at 4°C overnight. Sections were then washed 3 times with PBS, and incubated with streptavidin-horseradish peroxidase-conjugated anti-rabbit or -mouse immunoglobulin G for 30 min at room temperature. Finally, sections were incubated with H_2_O_2_-diaminobenzidine at room temperature until the desired intensity of stain had developed. All sections were counterstained with hematoxylin, dehydrated, and mounted to slides.

### Semi-quantitative Evaluation of Immunohistochemical Staining

In the analysis of IHC-stained slides, visible brown granules in the cytoplasm were identified as positive OLC1 staining. The staining intensity and extent of each specimen was reviewed. The staining intensity was rated as negative (0), bordering (1), weak (2), or strong (3), while the staining extent was rated based on the percentage of positive cells in the field. Samples with no stained cells were rated as 0, those with <25% were rated 1, those with 25%–50% were rated 2, and those with >50% of cells stained were rated as 3. The results of the staining intensity and extent gave an overall staining score. The samples in which the overall score was 0 were marked as (−), those that scored 1 to 2 marked as (±), 3 to 4 labelled (+), and 5 to 6 marked as (++). All slides were blindly labeled and scored by two independent pathologists.

### Statistical Analysis

All data represented at least three independent experiments and were statistically evaluated by Pearson chi-square test or Student’s *t* test with the statistical analysis software SPSS version 19.0 (IBM). Results are expressed as mean ± SEM, and *P* values less than 0.05 were considered to be statistically significant.

### Cell Extraction and Western Blot

Cell extraction and western blotting analyses were performed as previously described [26]. Caspase-3 (sc-7148), Bcl-2 (sc-509), and ß-actin (sc-8432) antibodies were purchased from Santa Cruz Biotechnology. All experiments were repeated three times.

### RT-PCR

Total RNA was isolated from cells using Trizol reagent (Invitrogen) following the manufacturer’s instructions, and total RNA was reverse-transcribed as described (Invitrogen). The sequences of the RT-PCR primers for OLC1 were: 5′-ACAGTGGGAGAGAGCACGTT-3′ (forward primer); 5′-GCACCTTGTCCTTTCTCTGC-3′ (reverse primer). These primers resulted in a PCR product that was 435 bp in length. For GAPDH, the primers were as follows: 5′-GCTGAGAACGGGAAGCTTGT-3′ (forward primer); 5′-GCCAGGGGTGCTAAGCAG-3′ (reverse primer), and they resulted in a PCR product of 299 bp.

### Cell Growth

Cancer cells in the exponential growth phase were digested with trypsin, suspended in culture medium containing 10% fetal bovine serum, and then seeded (2×10^4^ cells per 35-mm plates) in triplicate. For each plate, the cells were counted on days 1, 2, 3, 4, and 5, and the growth curves were plotted. All experiments were repeated three times.

### Colony Formation Assay

Cells were plated at a density of 1×10^3^ cells per well, in triplicate, on 6-well plates. After 14 days in a humidified 5% CO_2_ incubator at 37°C, the plates were washed with PBS, and the cells were fixed in cold methanol and stained with 0.5% crystal violet. Colonies with >50 cells were counted, and all experiments were repeated three times.

### DAPI Staining

Cells were trypsinized while in their exponential growth phase, suspended in culture with 10% fetal bovine serum, plated on to 30 mm plates, and incubated for 24 hr maintained at 37°C in a humidified 5% CO_2_ incubator. Cells were then treated with different doses of CDDP (cis-dichlorodiamine platinum, Haosen Pharmaceutical, Inc, Jiangsu, China). Cells were fixed with methanol, and nuclei were stained with 0.1 µg/mL DAPI (4′,6′-diamidino-2-phenylindole hydrochloride, Sigma). Cells with condensed nuclei when DAPI staining was visualized under a fluorescent microscope were deemed to be apoptotic.

## Results

### The Expression of OLC1 Protein was Progressively Increased in the Different Stages of ESCC

**Figure 1:**
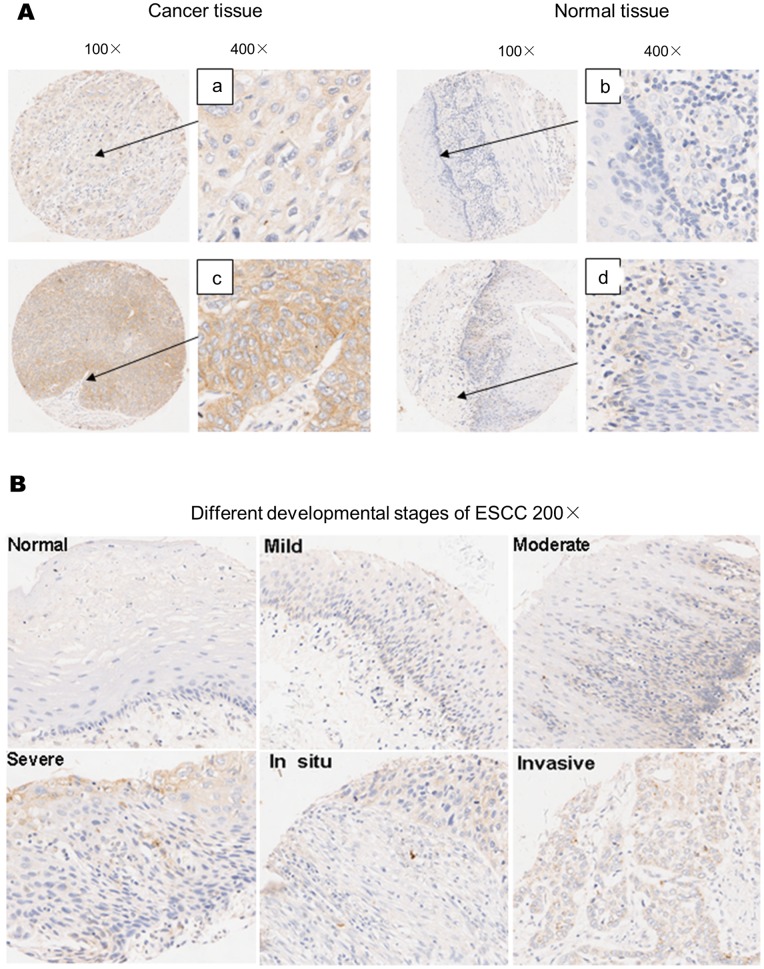
Expression of OLC1 in normal esophageal epithelium, dysplasia, and ESCC, as assessed by immunohistochemistry. (A) Images of representative IHC staining of OLC1 in human ESCC and paired adjacent normal tissue, magnified 100- and 400-fold, as indicated. OLC1 staining in the cytoplasm of ESCC tissue was positive (A–a) and strongly positive (A–c), but the paired adjacent normal tissues showed no or faint OLC1 staining (A–b and A–d). (B) The correlation of OLC1 protein expression with the developmental stages of human ESCC. Representative images of OLC1 staining in normal adjacent, mild dysplasia, moderate dysplasia, severe dysplasia, carcinoma in situ, and invasive carcinoma tissues (x200 magnification).

To detect the expression of OLC1 expression in human ESCC, 214 paired ESCC specimens were assessed by IHC staining followed by chi-squared analysis. The tumor samples all exhibited cytoplasmic staining of OLC1 (Figure 1A–a and 1A–c), but the paired adjacent normal tissues showed no or faint cytoplasmic staining (Figure 1A–b and 1A–d). Immunohistochemical analysis showed that OLC1 was overexpressed in 145 out of 214 (67.8%) human ESCC, compared with only 59 of 214 (27.57%) paired adjacent normal tissues (*P*<0.001). However, we found no significant differences in OLC1 expression between T3N0M0 (63.46%, 66/104) and T3N1M0 (70.91%, 78/110) in human ESCC tissues (*P* = 0.394) (Table 1).

**Table 1:** The relationship between OLC1 expression and patient clinicopathological characteristics.

	Expression of OLC1 protein			Total Patients	Percent positive (%)	*P* value*
	(−)(±)#	(+)#	(++)#			
**General**						
Normal Adjacent tissue	155	51	8	214	27.57	
Tumor tissue	69	115	30	214	67.80	<0.001
**Gender**						
Male	54	93	25	172		
Female	16	21	5	42		>0.05
**Age**						
<60	41	68	14	123		
≥60	29	46	16	91		>0.05
**TNM Stage**						
T3N0M0	38	54	12	104	63.46	
T3N1M0	32	60	18	110	70.91	>0.05

# (−) = no staining; (±) = background or faint staining; (+) = positive staining; (++) = intense staining. Samples marked (+) or (++) were considered positive for OLC1 protein expression.

*Significance was determined by Pearson chi-square test.

An independent set of tissue sections taken from 28 ESCC patients were used to verify the correlation between OLC1 expression and the different stages of tumorigenesis of human ESCC using IHC. As shown in Figure 1B, mild/moderate dysplasia (Mi-DYs/Mo-DYs) samples showed weak OLC1 staining, while severe dysplasia(S-DYs)/carcinoma in situ (CIS), and invasive carcinoma tissues showed strong cytoplasmic staining. In contrast, normal tissues were negative for OLC1 cytoplasmic staining. OLC1 overexpression was detected in 35% (10/28) of mild/moderate dysplasia specimens, but was significantly upregulated in 66% (22/33) of severe dysplasia and carcinoma in situ tissues (*P*<0.05). The OLC1expression in invasive carcinoma samples was 71% (22/28), while 16% (4/25) positive staining was observed in normal tissues (*P*<0.05) (Table 2).

**Table 2:** OLC1 expression in esophageal samples with different lesions.

	Expression of OLC1 protein			Total cases	Percent Positive (%)
Developmental stages	(−)(±)#	(+)#	(++)#		
Normal adjacent tissues	21	4	0	25	16
Mi-DYS/Mo-DYS	18	7	3	28	35
S-DYs/CIS	11	15	7	33	66
Invasive Carcinoma	6	12	10	28	71

# (−) = no staining; (±) = background or faint staining; (+) = positive staining; (++) = intense staining. Samples marked (+) or (++) were considered positive for OLC1 protein expression.

Significance was determined by Pearson chi-square test. S-DYs/CIS *vs.* Normal adjacent tissues, *P*<0.05; Invasive carcinoma *vs.* Normal adjacent tissues, *P*<0.05; Mi-DYS/Mo-DYS *vs.* Normal adjacent tissues, *P*>0.05; S-DYs/CIS *vs.* Mi-DYs/Mo-DYs, *P*<0.05; Invasive carcinoma *vs.* Mi-DYs/Mo-DYs, *P*<0.05; Invasive carcinoma *vs.* S-DYs/CIS, *P*>0.05.

### Overexpression of OLC1 Promotes the Growth and Colony Formation of ESCC Cells

**Figure 2:**
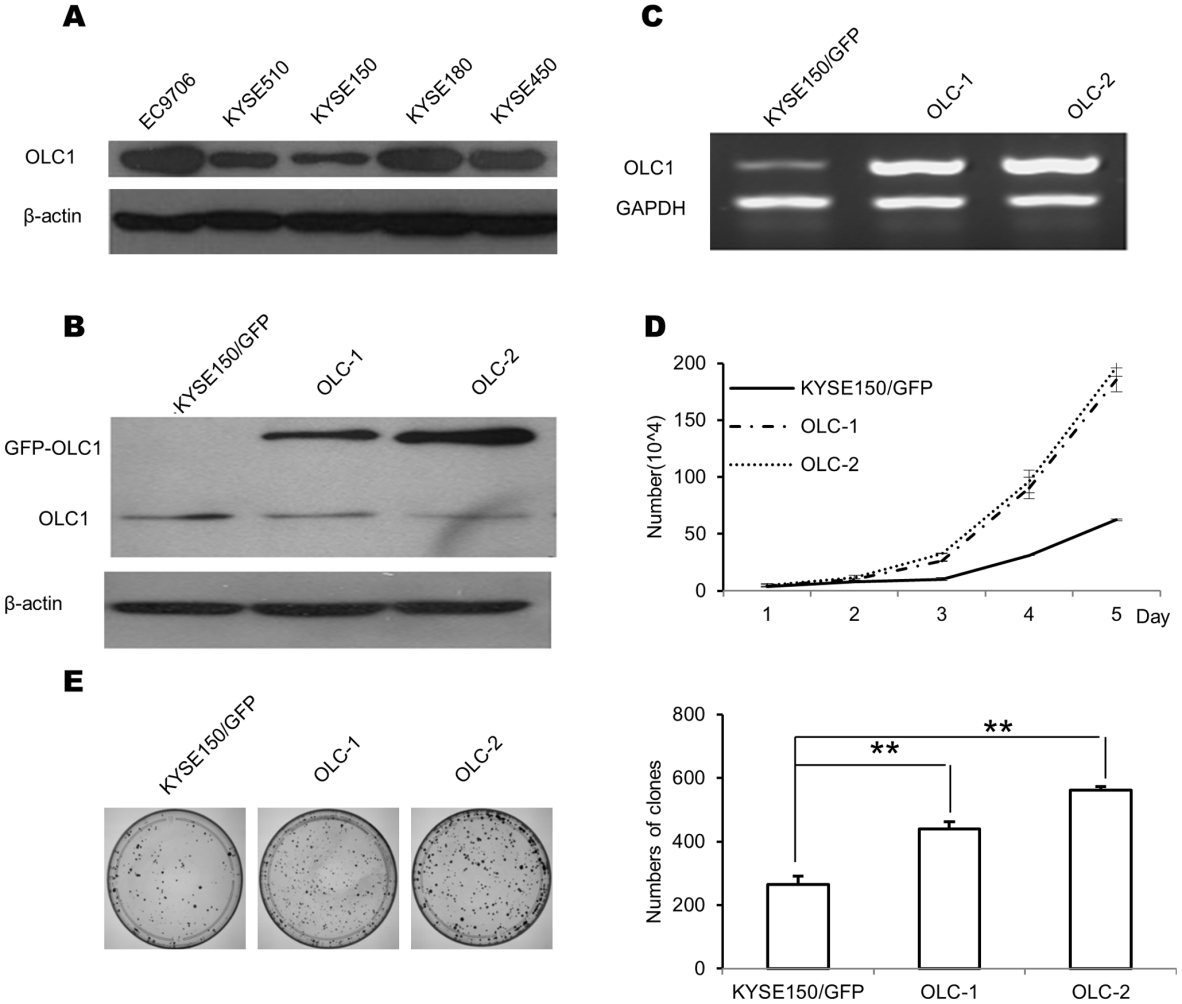
Construction of stable OLC1-overexpressing cell lines, and the effects of OLC1 overexpression on KYSE150 cells. (A) To investigate OLC1 expressions among ESCC cell lines (EC9706, KYSE 510, KYSE 150, KYSE450 and KYSE180), the relative protein levels of OLC1 from these cell lines were examined by western blotting analysis. (B) KYSE150 cells were transfected with pEGFP-N1-OLC1 and pEGFP-N1, and stable, positive clones were selected using with G418 (400 µg/mL) from candidate clones. Two positive clones were designated as OLC-1 and OLC-2, the null control colony was designated KYSE150/GFP. Confirmed by western blotting, the expressed GFP-OLC1 fusion protein (68 kDa) was detected in OLC-1 and OLC-2, but not in KYSE150/GFP cells (C) mRNA levels of OLC1 in OLC-1, OLC-2 and KYSE150/GFP cells, assessed by RT-PCR. (D) Cell proliferation curves were calculated after counting the numbers of OLC-1, OLC-2 and KYSE150/GFP cells for 5 days. (E) The numbers of colonies of each cell line were counted, and then compared. All experiments were repeated three times. Significance was determined by the Student’s t test. * = P<0.05; ** = P<0.001.

To evaluate the role of OLC1 expression in ESCC, the cell line KYSE150, which expresses a relatively low level of endogenous OLC1 protein (Figure 2A), was used. KYSE150 cells were transfected with pEGFP-N1-OLC1 or pEGFP-N1, and the stable OLC1-overexpressing cell line was named KYSE150/GFP-OLC1, while the null control clone was named KYSE150/GFP. Two OLC1 overexpressing clones (OLC-1 and OLC-2) and one null control clone were used for further analyses. As expected, the GFP-OLC1 fusion protein (68 kDa) was detected in OLC-1 and OLC-2, but not in control cells (Figure 2B). Consistent with these data, RT-PCR analysis showed a strong band in OLC-1 and OLC-2 cells, but only a weak band in KYSE150/GFP cells (Figure 2C). We then used the 3 cell lines to assess the effects of OLC1 overexpression on the characteristics of KYSE150 cells using cell proliferation and colony formation assays. OLC-1 and OLC-2 grew significantly faster than KYSE150/GFP (P<0.05; Figure 2D). In addition, the number of colonies formed by OLC-1 and OLC-2 cells were 440.33±22.12 and 562±11.53, which were significantly higher than that those formed by control cells (264.67±26.27; P<0.001; Figure 2E).

### Apoptosis Inhibited by OLC1 Overexpression is Associated with Attenuated Caspase-3 Activation and Increased Bcl-2 Stability

**Figure 3:**
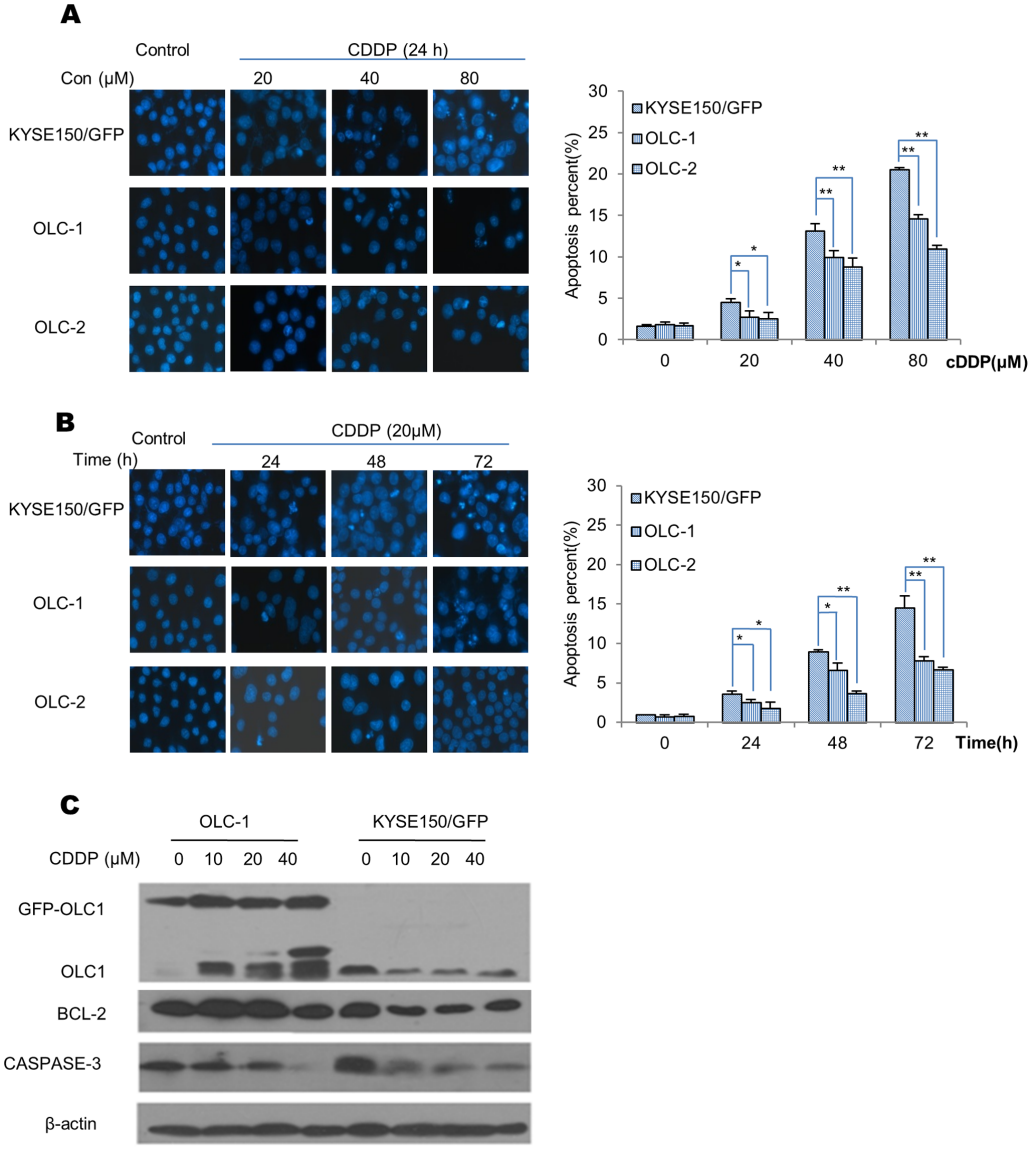
The effects of OLC1 overexpression on CDDP-induced apoptosis. (A) Cell lines were treated with CDDP at 0, 20, 40, or 80 µM for 24 h, followed by DAPI staining and analysis by fluorescent microscopy and photography. The total number of the apoptotic cells was counted, and the percentage of apoptotic cells was calculated. (B) Cells were treated with 20 µM CDDP for different time periods (including 0, 24, 48, 72 h) as indicated, followed by DAPI staining. (C) Cells were treated with different doses of CDDP (including 0, 10, 20, 40 µM) for 24 h, and the effects of OLC1 on the expression of the apoptosis-related proteins caspase-3 and Bcl-2 was assessed by western blotting. Significance was determined by the Student’s *t* test. * = *P*<0.05; ** = *P*<0.001.

We further investigated the role of OLC1 overexpression on CDDP-induced apoptosis in OLC-1 and OLC-2 and KYSE150/GFP. CDDP induced apoptosis in a dose-dependent manner in the 3 cells. However, the increases were lower in OLC-1 and OLC-2 compared with KYSE150/GFP (Figure 3A). When the cells were treated with 20 µM CDDP, apoptosis increased over time in the 3 cell lines. The percentages of apoptotic OLC-1 and OLC-2 cells were also lower than their control cells (Figure 3B). Taken together, these results suggest that the overexpression of OLC1 inhibited CDDP-induced apoptosis in a time- and dose-dependent manner.

Next, western blotting was used to assess the expression of OLC1, Bcl-2, and caspase-3 after cells had been treated with CDDP. As the concentration of CDDP increased (Figure 3C), a concurrent fall in caspase-3 pro-band was seen in both cell lines. However, less pro-band was seen in KYSE150/GFP compared with OLC-1 cells. In addition, Bcl-2 was more stable in OLC-1 cells compared with KYSE150/GFP after treatment with CDDP. These data suggest that OLC1 overexpression inhibits apoptosis, by stabilizing Bcl-2.

### Stable Knockdown of Endogenous OLC1 Inhibits Cell Growth and Increases CDDP-induced Apoptosis

**Figure 4:**
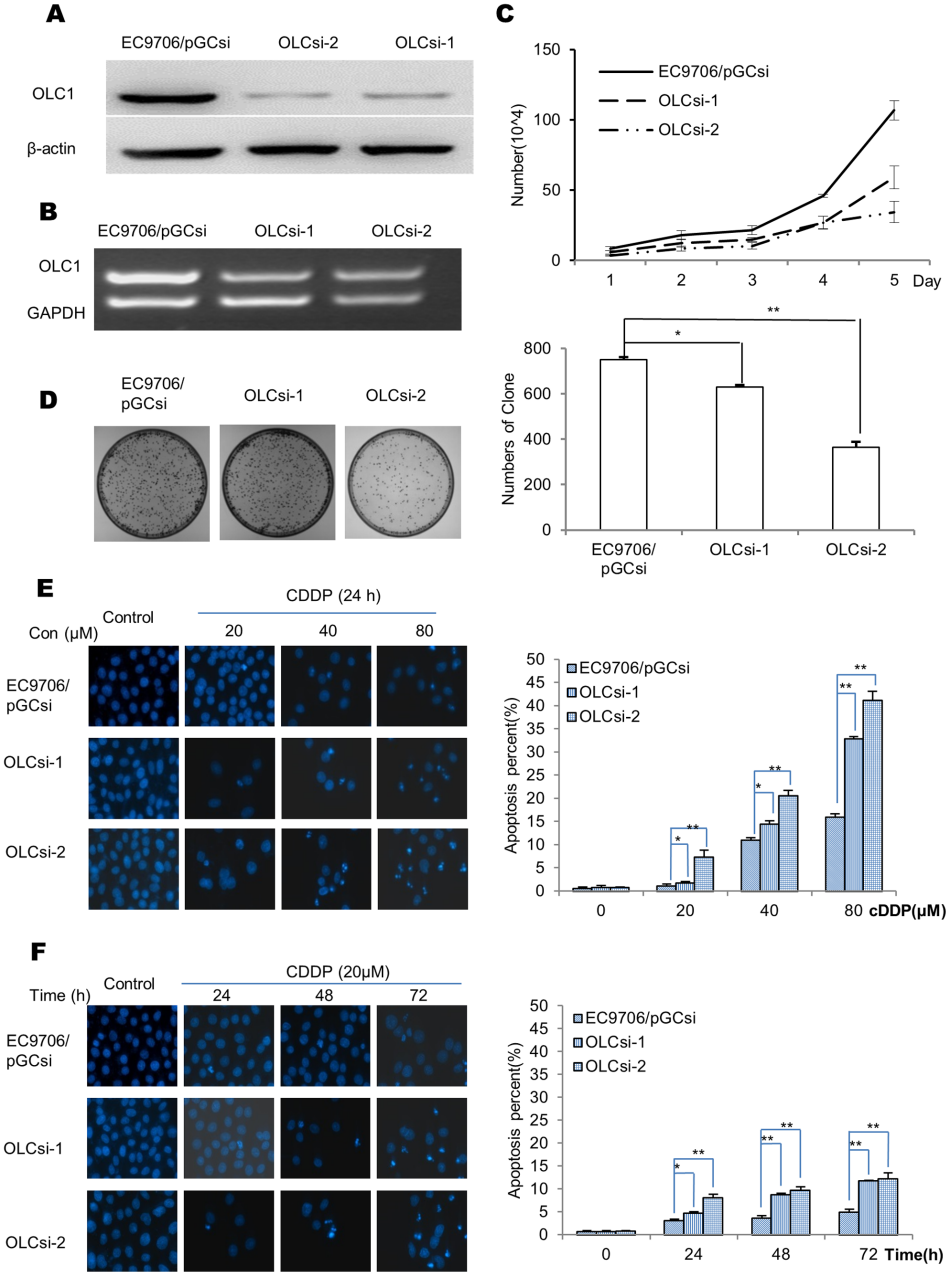
OLC1-interfering cell lines and their differential effects on cell growth and apoptosis in response to CDDP treatment. (A) EC9706 cells were transfected with PGCsi-U6/neo/GFP-control or PGCsi-U6/neo/GFP-OLC1. The 2 stable OLC1-interferencing cell lines and paired control were verified by western blotting, and named OLCsi-1, OLCsi-2 and EC9706/pGCsi, respectively. (B) Evaluation of the mRNA levels of OLC1 in OLCsi-1, OLCsi-2 and EC9706/pGCsi cells, assessed by RT-PCR. (C) The cell proliferation curve was calculated from cell counting data over 5 days. (D) Graph showing the total numbers of colonies of OLCsi-1, OLCsi-2 and EC9706/pGCsi cells. (E) OLCsi-1, OLCsi-2 and EC9706/pGCsi cells were treated with CDDP at concentrations of 0, 20, 40, 80 µM for 24 h, and apoptosis was assessed by DAPI staining. The graph shows the percent of apoptotic cells from each cell line. Significance was determined by the Student’s *t* test. * = *P*<0.05; ** = *P*<0.001.

EC9706 cells, which express relatively higher levels of endogenous OLC1 (Figure 2A), were transfected with PGCsi-U6/neo/GFP-control or PGCsi-U6/neo/GFP-OLC1. We designated the 2 stable OLC1 knockdown clones as OLCsi-1 and OLCsi-2, and the negative control as EC9706/pGCsi. As expected, the mRNA and protein levels of OLC1 were reduced in OLCsi-1 and OLCsi-2 cells compared with EC9706/pGCsi, as assessed by western blotting and RT-PCR, respectively (Figure 4A and 4B). OLCsi-1 and OLCsi-2 cells grew slower (P<0.05) and formed fewer colonies (*P<*0.001) than EC9706/pGCsi cells (Figure 4C and 4D).

We further evaluated the effects of OLC1-interference on apoptosis with DAPI staining. CDDP-induced apoptosis in EC9706/pGCsi cells was markedly lower than in OLCsi-1 and OLCsi-2 cells, and they were all in a dose- and time-dependent manner (Figure 4E and 4F).

## Discussion

ESCC is usually diagnosed when the tumor is at an advanced stage, and so frequently has poor prognosis. It is therefore important to identify new candidate markers to facilitate the early diagnosis and treatment of ESCC. The abnormal expression of several proteins, including p63 [27]–[29], SMAD6 [30], SMAD7 [30], [31], FHIT [3], [32], [33], and Annexin I [34], [35], has been reported in the early stages of ESCC.

In this study, several experiments revealed that the expression of OLC1 is progressively increased during the development of esophageal dysplasia and ESCC. This suggests that elevated expression of OLC1 occurs before tumor metastasis, and that high OLC1 levels are probably maintained until ESCC metastasizes. In addition, immunohistochemical analysis of 214 ESCC specimens at the T3 stage, which show no significant differences in OLC1 expression between the non-lymph node (T3N0M0) and lymph node metastasis groups (T3N1M0) (Table 1), support this conclusion. Alteration of OLC1 expression could therefore be a critical event during the developmental of human esophageal carcinoma. Yuan et al. provided evidence that OLC1 overexpression may be involved in human lung carcinogenesis, especially during the early stages, which is consistent with our IHC data from ESCC patients. This suggests that the dysregulation of OLC1 may contribute to the development of human ESCC.

ESCC is one of the most common malignant neoplasms, and apoptotic dysfunction plays an important role in its development. Accordingly, a better understanding of the molecular mechanisms by which OLC1-induces the malignant cellular phenotypes of ESCC is required. Here, we provided novel evidence that dysregulation of OLC1 expression significantly alter the cell proliferation and apoptosis induced by cytotoxic agents. We observed that OLC1 overexpressing cells had increased proliferation and resistance to cisplatin-induced apoptosis compared with control cells. Conversely, we constructed OLC1-knockdown cell lines in EC9706 cells using siRNA, and stable knockdown of endogenous OLC1 inhibited cell growth and sensitized cells to CDDP-induced apoptosis. In addition, OLC1 overexpression led to reduced caspase-3 activation, and enhanced stability of Bcl-2. Caspase-3 is an important executioner caspase whose cleavage and activation leads to apoptosis executed by both death receptor-mediated or mitochondrial-dependent pathways [26], [36]–[40]. Bcl-2, an anti-apoptotic protein, suppresses apoptosis in several ways, including inhibiting cytochrome c release from the mitochondria [41]–[45]. Our findings suggest that OLC1 overexpression suppresses cellular apoptosis, possibly by interfering with caspase-3 activation and enhancing Bcl-2 stability. Taken together, these results suggest that the dysregulation of OLC1 disrupts the delicate balance between cell growth and apoptosis. Our results therefore help to explain why abnormalities in OLC1 expression contribute to a malignant cellular phenotype in ESCC.

Yuan et al. transformed NIH3T3 cells with *OLC1* and injected them into nude mice to assess the tumorigenicity of OLC1 [19]. Fibrosarcomas were detected in all animals that were inoculated with OLC1-expressing NIH3T3 cells, but not in the control groups injected with the parental or empty-vector transfected cells. As a novel potential oncogene, our results reveal that OLC1 is a cell cycle-dependent protein that may be involved with ubiquitin-dependent degradation [46]. OLC1 also plays a role in cytokinesis [47]–[51]. Taken together, these data suggest that OLC1 may play an important role in regulating the cell cycle, and ultimately cellular growth and apoptosis. However, more studies are needed to explore the underlying mechanisms of OLC1 dysregulation in esophageal tumorigenesis.

In conclusion, we report that OLC1 is overexpressed in human ESCC; OLC1 abnormalities may contribute to the development of human ESCC and have some important clinical significance.
